# Outer Retinal Layer Thickness Changes in White Matter Hyperintensity and Parkinson's Disease

**DOI:** 10.3389/fnins.2021.741651

**Published:** 2021-09-14

**Authors:** Yitian Zhao, Jinyu Zhao, Yuanyuan Gu, Bang Chen, Jiaqi Guo, Jianyang Xie, Qifeng Yan, Yuhui Ma, Yufei Wu, Jiong Zhang, Qinkang Lu, Jiang Liu

**Affiliations:** ^1^Cixi Institute of Biomedical Engineering, Ningbo Institute of Materials Technology and Engineering, Chinese Academy of Sciences, Ningbo, China; ^2^Zhejiang International Scientific and Technological Cooperative Base of Biomedical Materials and Technology, Ningbo Institute of Materials Technology and Engineering, Chinese Academy of Sciences, Ningbo, China; ^3^Zhejiang Engineering Research Center for Biomedical Materials, Ningbo Institute of Materials Technology and Engineering, Chinese Academy of Sciences, Ningbo, China; ^4^School of Mechanical Engineering, Southwest Jiaotong University, Chengdu, China; ^5^The Affiliated People's Hospital of Ningbo University, Ningbo, China; ^6^Department of Computer Science and Engineering, Southern University of Science and Technology, Shenzhen, China

**Keywords:** white matter hyperintensities, Parkinson's disease, outer retinal layers, OCT images, deep learning

## Abstract

**Purpose:** To investigate the thickness changes of outer retinal layers in subjects with white matter hyperintensities (WMH) and Parkinson's Disease (PD).

**Methods:** 56 eyes from 31 patients with WMH, 11 eyes from 6 PD patients, and 58 eyes from 32 healthy controls (HC) were enrolled in this study. A macular-centered scan was conducted on each participant using a spectral-domain optical coherence tomography (SD-OCT) device. After speckle noise reduction, a state-of-the-art deep learning method (i.e., a context encoder network) was employed to segment the outer retinal layers from OCT *B*-scans. Thickness quantification of the outer retinal layers was conducted on the basis of the segmentation results.

**Results:** WMH patients had significantly thinner Henle fiber layers, outer nuclear layers (HFL+ONL) and photoreceptor outer segments (OS) than HC (*p* = 0.031, and *p* = 0.005), while PD patients showed a significant increase of mean thickness in the interdigitation zone and the retinal pigment epithelium/Bruch complex (IZ+RPE) (19.619 ± 4.626) compared to HC (17.434 ± 1.664). There were no significant differences in the thickness of the outer plexiform layer (OPL), the myoid and ellipsoid zone (MEZ), and the IZ+RPE layer between WMH and HC subjects. Similarly, there were also no obvious differences in the thickness of the OPL, HFL+ONL, MEZ and the OS layer between PD and HC subjects.

**Conclusion:** Thickness changes in HFL+ONL, OS, and IZ+RPE layers may correlate with brain-related diseases such as WMH and PD. Further longitudinal study is needed to confirm HFL+ONL/OS/IZ+RPE layer thickness as potential biomarkers for detecting certain brain-related diseases.

## 1. Introduction

White matter, gray matter and substantia nigra are three important components of the central nervous system. There exists strong evidence showing that white matter hyperintensities (WMH) is an important clinical markers of several brain diseases, such as stroke, dementia, in both cross-sectional and longitudinal studies (Balakrishnan et al., 2021; Song et al., 2021). Degeneration of the nigra cells leads to a decrease in dopamine neurons, which causes Parkinson's disease (PD). Therefore, efficient detection of hyperintense lesions in cerebral white matter and substantia nigra damage is of potential importance in the further study of diseases of the brain.

White matter hyperintensities (WMH) is the area of brain tissue that show up as increased brightness in T2-weighted magnetic resonance imaging (MRI) (Wardlaw et al., 2013). In clinical practice, a structural MRI (Zhuang et al., 2017) can be used for quantitative calculation of the volume of brain tissue for the diagnosis of WMH, while diffusion tensor imaging (DTI) (Etherton et al., 2017, 2019) is used to detect early micro-structural changes in white matter. The arterial spin labeling (ASL) (Van Dalen et al., 2016) method employed blood as an endogenous tracer for 3D flair scanning, in which the WMH volume was inversely proportional to the regional perfusion level. In addition, carotid ultrasound, cerebral angiogram, and echocardiogram have also been used to identify WMH.

PD is a neurodegenerative disorder, mainly affected by dopaminergic neurons in a specific area of the brain called the substantia nigra (Shen et al., 2021). Currently, PD is usually diagnosed based on motor symptoms (Oh et al., 2018). Common lab tests from blood, urine, or cerebrospinal fluid (CSF) can be used to detect PD. Meanwhile, brain imaging techniques [e.g., MRI, and Positron Emission Tomography (PET)] have also been proved as highly sensitive tools for identifying PD. However, most detection methods for WMH and PD are cost-intensive and invasive (usually involving the injection of a dye into the patient to make blood vessels stand out more prominently). It's necessary to explore a cheap and non-invasive alternative detection method.

It is commonly accepted that there are various relationships between eye and brain (Baker et al., 2008). The retina develops from the diencephalon in the embryonic period. Retinal vessels possess similar anatomic and physiological characteristics to cerebral vessels. There is also a blood-retinal barrier analogous to blood-brain barrier. In many chronic diseases, changes in retinal vessels may reflect, or even precede changes in cerebral vessels. Furthermore, the retina is the only organ whose vascular imaging can be acquired noninvasively *in vivo*. Therefore, considerable efforts have been made to investigate the progression of brain-related disorders based on retinal signs. Lindley et al. (2009) found that, lacunar stroke was more likely to produce retinal micro-vessel signs, such as focal arteriolar narrowing, arteriovenous nipping, generalized retinal arteriolar narrowing, small retinal arteriole to venule ratio, and retinal venular widening, compared with other stroke subtypes. Optical coherence tomography (OCT) is an efficient cross-sectional bioimaging modality that has become indispensable to retinal examination. Wang et al. (2014) employed spectral-domain OCT to detect localized retinal nerve fiber layer defects (RNFLDs) and confirmed that they were strongly associated with acute ischemic stroke, or prior cerebral stroke. Moreno-Ramos et al. (2013) revealed a significant decrease in the thickness of the retinal nerve fiber layer (RNFL) in AD, PD, and Lewy body dementia compared to normal controls, but the thickness changes were not statistically significant between these three diseases. Thomson et al. (2015) conducted a meta-analysis of RNFL change in dementia and concluded that the RNFL thickness had the potential to distinguish AD, or mild cognitive impairment (MCI) from healthy controls (HC). Thinning of the ganglion cell-inner plexiform layer (GC-IPL) may be found at the preclinical stage of AD (Fyfe, 2018; Cheung et al., 2019; Ma et al., 2021). Bulut et al. (2018) analyzed retinal vascular density (RVD), the foveal avascular zone (FAZ), choroidal thickness (CT), and outer retinal and choroidal flow rate in AD patients and HC via OCT angiography (OCTA) imaging, and highlighted the potential role of those metrics in the early diagnosis of AD. Ma et al. (2018) studied potential correlations between PD and retinal changes using OCT, finding that the average RNFL thickness, total macular thickness and MV were significantly decreased in PD patients compared to HC. Matlach et al. (2018) carried out some correlation analysis, but ruled out macular inner retinal thickness and peripapillary RNFL thickness to be effective biomarkers of PD patients. Chrysou et al. (2019) performed a relatively detailed meta-analysis of retinal changes in PD, and confirmed significant thinning of peripapillary RNFL and combined GC-IPL in PD.

**Figure 1:**
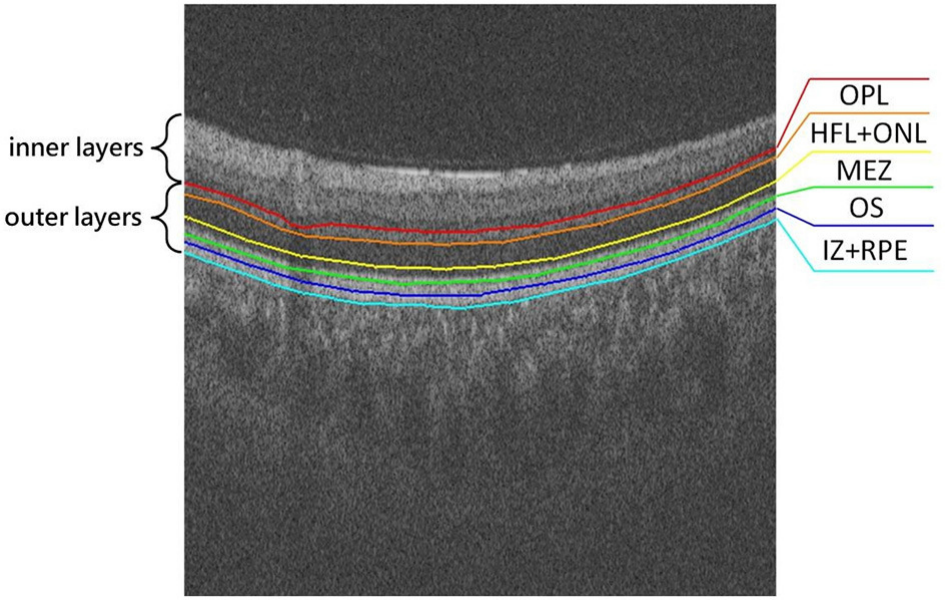
Illustration of the outer retina layered structure, from top to bottom: outer plexiform layer (OPL), Henle fiber layer and outer nuclear layer (HFL+ONL), myoid and ellipsoid zone (MEZ), photoreceptor outer segments (OS), interdigitation zone and retinal pigment epithelium/Bruch complex (IZ+RPE).

For retinal structural changes associated with AD/PD/dementia, most prior work focused on the thickness of the RNFL, GCL, and/or GC-IPL. However, the retina is usually composed of inner layers and outer layers, as shown in Figure 1. The inner retinal layers include the RNFL, GCL, inner plexiform layer (IPL), and inner nuclear layer (INL), while the outer retinal layers comprise the outer plexiform layer (OPL), the Henle fiber layer and outer nuclear layer (HFL+ONL), the myoid and ellipsoid zone (MEZ), the photoreceptor outer segments (OS), the interdigitation zone and the retinal pigment epithelium/Bruch complex (IZ+RPE). On one hand, RPE layer in the outer retina may produce levodopa, a precursor to dopamine (McKay et al., 2006). The degeneration of dopaminergic neurons is an important feature of Parkinson's Disease. On the other hand, the INL+HFL and OS layers in the outer retina contain axons and dendrites of photoreceptor cells, respectively (Lujan et al., 2011). The NFL containing ganglion cell axons, and the IPL containing ganglion cell dendrites were confirmed to be thinner in WMH patients. To the best of our knowledge, the study of associations between thickening in any of the outer retinal layers and brain-related diseases has remained relatively unexplored. In this study, we use a deep learning model to segment the outer retinal layer automatically from OCT images, and measure the thickness of each layer. We then utilize these thickness indicators to investigate outer retinal layer alterations within the PD, WMH and HC groups, respectively, in order to find a potential retina-related index for the auxiliary diagnosis of these diseases.

## 2. Experiments

Ethical approval for all experiments was provided by the institutional ethics committees, and written informed consent was obtained from each individual in accordance with Declaration of Helsinki.

### 2.1. Data Collection

#### 2.1.1. Healthy Controls

There were 32 participants of similar age and sex to the WMH and PD patients. HC were subjects without cognitive impairment. Any subject with any of certain ocular diseases (such as glaucoma, macular degeneration, cataract, or high myopia) or with a systematic/neurological disease (such as diabetes, hypertension or multiple sclerosis) or prior ocular surgery was excluded from this study.

#### 2.1.2. WMH and PD Participants

In total 56 eyes from 31 WMH patients and 11 eyes from 6 PD patients were enrolled in this study. Demographic data (age and sex) of WMH and PD participants were obtained through self-reporting questionnaires. WMH cases were evaluated by MRI technique, using a spin density image to estimate the total volume of subcortical white matter signal abnormalities. Participants were divided into WMH and HC groups by their total Fazekas scores, according to the reference standards of the presence of white matter abnormalities (Fazekas et al., 1987).

PD patients were diagnosed according to the UK Parkinson's Disease Society Brain Bank Clinical Diagnostic Criteria (Hughes et al., 1992). To satisfy the diagnosis of bradykinesia, a patient should have at least one of the following symptoms: muscular rigidity, 4–6 Hz rest tremor, and postural instability that are not caused by primary visual, vestibular, cerebellar, or proprioceptive dysfunction.

### 2.2. OCT Image Acquisition

The RTVue XR Avanti SD-OCT system (Optovue Inc., Fremont, California, USA) was employed to perform a macular-centered OCT scan for each participant. Each scanned volume comprised 400 B-scans with a resolution of 400 × 400 × 640 pixels, and covered a field of view of 6.00 × 6.00 × 1.99 *mm*^3^.

### 2.3. Methods

A senior ophthalmologist manually annotated 160 OCT images for our validation. The labeled mask containing 5 layers of outer retinal sublayer based on our internally developed labeling software. Due to the characteristics of coherent light imaging, OCT is susceptible to coherent/speckle noise, resulting in significant degradation in spatial resolution and image quality. In order to overcome these quality defects, a multi-frame fusion-based super-resolution reconstruction algorithm (Yan et al., 2020) was adopted as a preprocessing step before segmentation to improve the resolution of the OCT images. A deep learning-based algorithm (i.e., context encoder network namely CE-Net Gu et al., 2019) was then employed to segment the outer retinal layers from the volumetric images (Please see Appendix in Supplementary Material and Figure 5 for more details). CE-Net was originally proposed for medical image segmentation specifically to overcome feature resolution reduction caused by consecutive pooling or convolution striding, and it has been proven to be effective in retinal OCT layer segmentation.

In the training stage, we input the super-resolution reconstructed OCT image which were cropped to 512 × 400 pixels automatically to the CE-Net, and calculate the Dice loss function (Crum et al., 2006) for the predicted map of the network output with the paired label. During the training, batch size was set to 4 and we adopt the SGD optimizer with a weight decay of 5e-4 to train the entire network end-to-end. The learning rate we set to 2e-4.

In this work, we first employed the CE-Net to detect the outer retinal layer boundaries, then we asked an ophthalmologist to review the automated detection results. The boundaries with poor segmentation performance were manual adjusted by using open source software ImageJ (https://imagej.nih.gov/ij/). Finally, thickness quantification of the outer retinal layers was performed on the basis of previous segmentation results. By calculating the thickness of the outer retinal layer for each B-scan of the same subject, the global thickness indicator of the corresponding person was obtained.

### 2.4. Statistical Analysis

The statistical analysis was performed using IBM SPSS Statistics v22.0 software (IBM Inc., Armonk, NY, USA). To compare the characteristics of subjects among groups, Student's *t*-tests and chi-square tests were performed for continuous variables and categorical ones, respectively. If the samples across each group meet the hypothesis of homogeneity of variance, the One-way ANOVA models is applied to analyze the thickness difference among three groups. Otherwise, the Kruskal Wallis test for independent samples was applied for continuous variables, and the chi-square test for cateforical variables. Furthermore, associations between incidence of disease and the thickness information of the retinal layers were assessed using multivariate logistic regression analysis, in which the models of the WMH and PD groups were adjusted for age and gender. *p* < 0.05 was regarded as statistically significant. The basic demographics of the subjects are shown in Table 1. There was no significant difference in either age or sex between groups.

**Table 1:** Basic characteristics of WMH, PD, and HC groups.

**Characteristics**	**WMH**	**PD**	**HC**	***P*_1_**	***P*_2_**
	**(*n* = 31)**	**(*n* = 6)**	**(*n* = 32)**		
Eyes (OD), n	56 (28)	11 (5)	58 (28)	N/A	N/A
Age, years	63.58 ± 7.12	62.67 ± 11.48	60.56 ± 5.72	0.239^◇^	0.492^◇^
Female, n	12	4	20	0.059^‡^	0.846^‡^

*OD represents the right eyes*.

*P_1_ and P_2_ denote the significance between WMH and HC, and PD and HC, respectively*.

*P^◇^ value was obtained by Student's t-test and P^‡^ value was obtained by chi-square test*.

### 2.5. Results

#### 2.5.1. Descriptive Statistics

**Figure 2:**
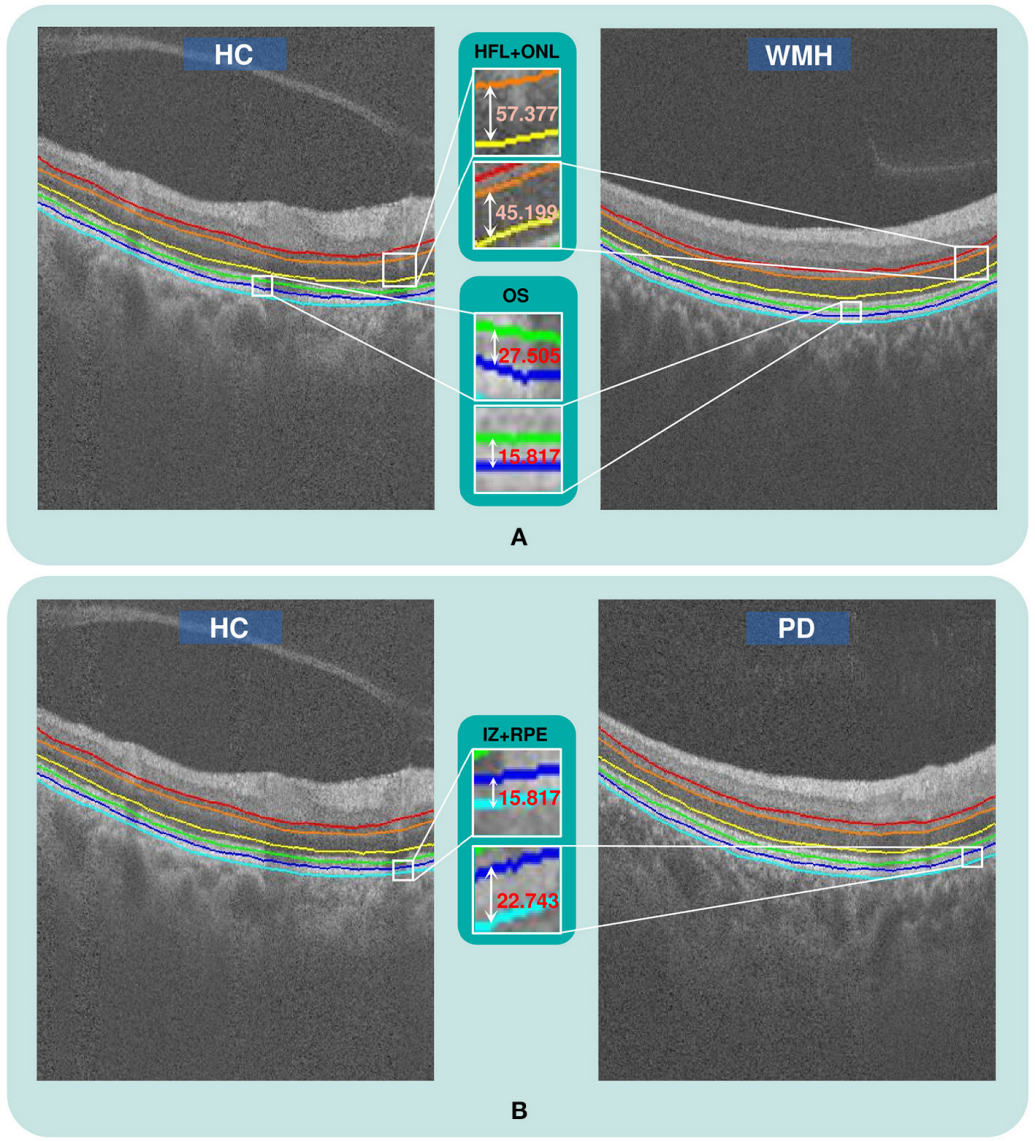
The qualitative comparison of outer retinal layer thickness. **(A)** The thickness of the HFL+ONL and OS layers in a WMH case is obviously reduced compared with a healthy subject from the HC group, **(B)** The thickness of the IZ+RPE layer in a PD case is obviously greater than in a HC case.

**Figure 3:**
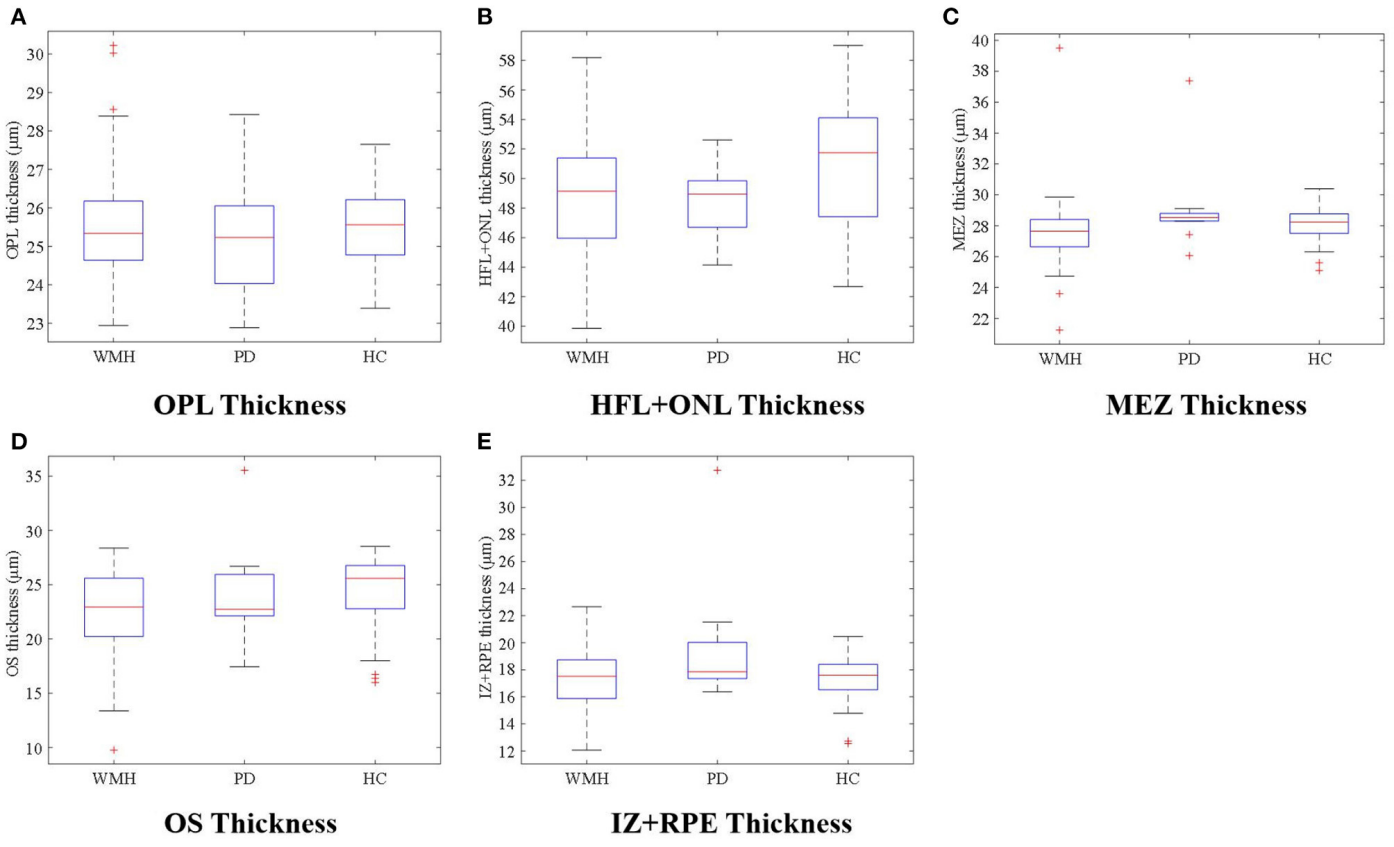
**(A–E)** illustrate thickness differences in the outer retina sublayers between the WMH, PD, and HC groups.

**Figure 4:**
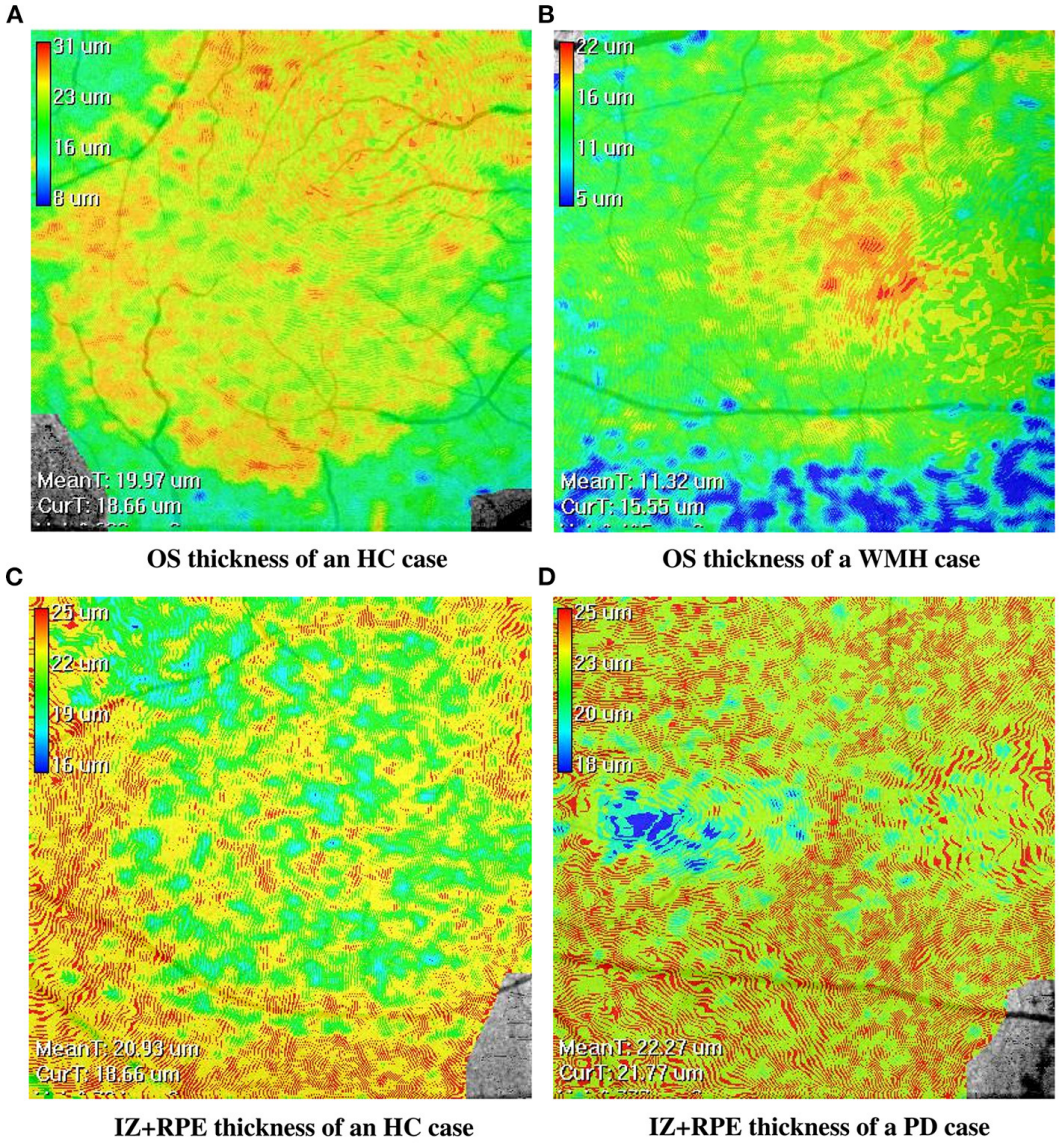
The en-face retinal layer thickness maps of four cases from the WMH, PD and HC groups, respectively. **(A,B)** show the thickness distribution difference of the OS layer between the HC and WMH groups. **(C,D)** show the thickness distribution difference of the IZ+RPE layer between the HC and PD groups.

The visual comparison results of outer retinal layer segmentation of 3 example images from different groups are shown in Figure 2. The box plots in Figure 3 illustrate median, 1st/3rd quartile and lower/upper values within 1.5 interquartile ranges of 5 outer retinal layers. To quantitatively evaluate disease correlation, thickness comparisons between the WMH, PD and HC groups are summarized in Table 2. In the joint thickness of HFL and OPL, there is a significant difference (*p* = 0.031) between the WMH, PD and HC groups, while the MEZ thickness also shows a significant difference between the three groups (*p* = 0.042). The OS thickness of the WMH group exhibits an obvious decrease compared with the PD and HC groups (*p* = 0.008). Moreover, it is worth mentioning that the mean thickness of IZ+RPE in the PD group was significantly thicker than that in the other two groups. However, due to small sample size, the variance is non-homogeneous, and there is no obvious significance in the nonparametric test. In Figure 4, we present the thickness differences between outer retinal layers of 3 cases from different groups.

**Table 2:** Thickness comparison among WMH, PD, and HC (μ*m*).

**Outer retinal layer**	**WMH (*n* = 56)**	**PD (*n* = 11)**	**HC (*n* = 58)**	***p***
OPL	25.542 ± 1.479	25.179 ± 1.578	25.553 ± 1.031	0.669
HFL+ONL	49.282 ± 4.689	48.474 ± 2.425	51.178 ± 4.213	**0.031**
MEZ	27.575 ± 2.224	29.607 ± 2.884	28.091 ± 1.116	**0.042**
OS	22.354 ± 4.059	24.096 ± 4.580	24.550 ± 3.190	**0.008**
IZ+RPE	17.290 ± 2.228	**19.619** **±** **4.626**	17.434 ± 1.664	0.268^*^

*p-value was obtained by ANOVA, and P*

* *represents the result of a nonparametric test (Kruskal Wallis test) applied when the sample variance was non-homogeneous. Bold values indicate statistical significance of P < 0.05*.

**Figure 5:**
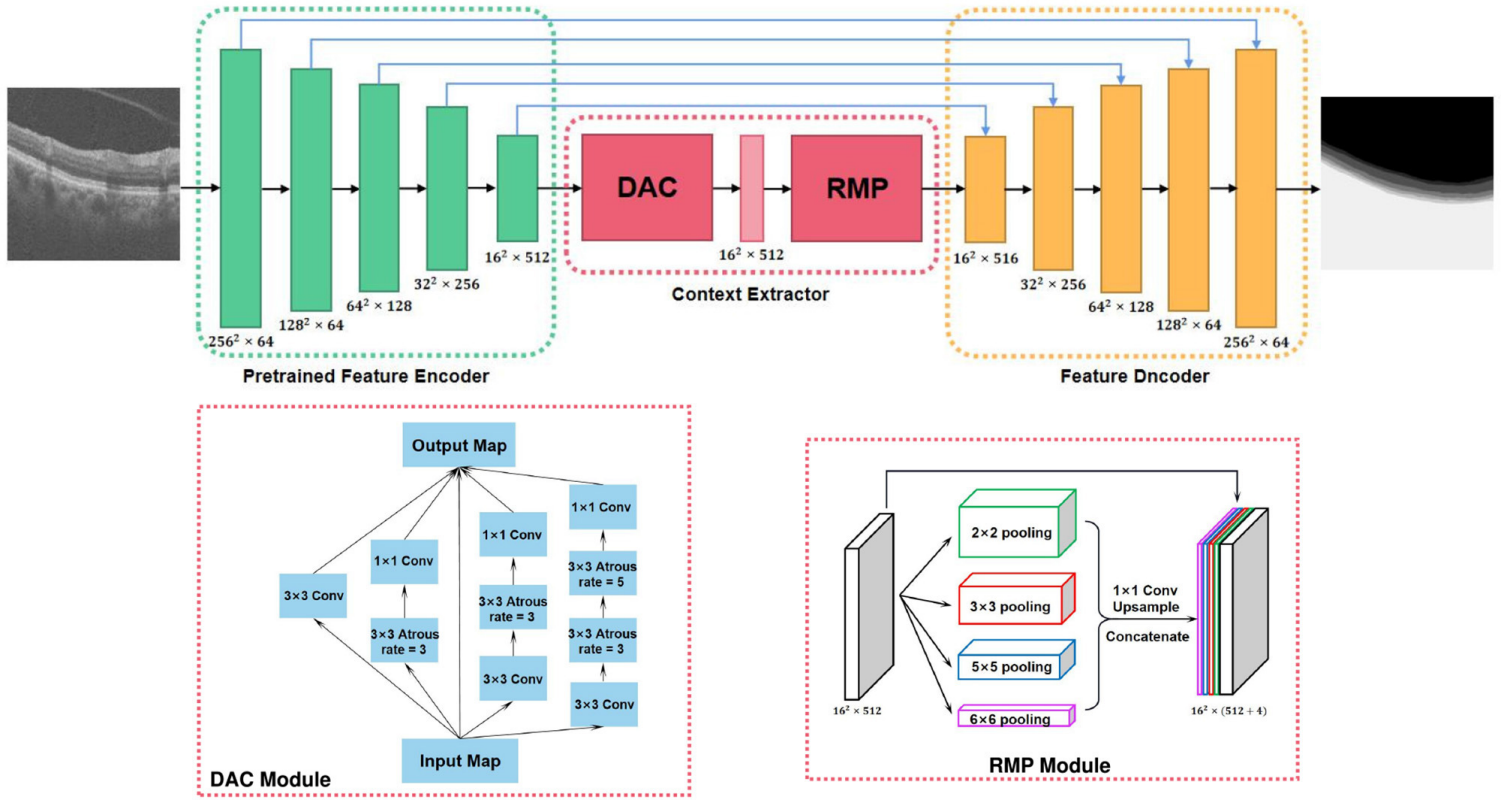
Overview of the context encoder network (CE-Net) (Gu et al., 2019).

#### 2.5.2. Logistic Regression Analysis

Some studies concluded that the outer retinal thickness was significantly correlated with age and sex. However, in our study we could not find a significant collinear relationship between these variables. Therefore, we took age, gender and thickness as independent variables, and took the HC group as the reference group for multiple logistic regression analysis with the PD and WMH groups, respectively. Considering the reliability of multivariate logistic regression over a small sample size, the demographic data was selected before enrolled as independent variables. Demographic variables with *p* < 0.2 in the univariate logistic regression were selected for adjustment in the multivariate logistic regression if the number of variables was more than one-tenth of the sample size. The results are shown in Table 3. Excluding the interference of age and gender, the thickness of the HFL+OPL and the OS layers in WMH patients were significantly reduced when compared to the HC group (*p* = 0.031 and *p* = 0.005, respectively). However, there was no significant difference in the thickness of the OPL, MEZ and IZ+RPE layer between these two groups. For the PD and HC groups, there was no statistically significant difference (*p* > 0.05) between PD patients and healthy people in the outer retinal layers.

**Table 3:** Results of multivariate logistic regression for demographic data.

**Outer retinal layer**	**WMH**			**PD**		
	**OR**	**95% CI**	***P*_1_**	**OR**	**95% CI**	***P*_2_**
OPL	1.022	0.758–1.380	0.885	0.791	0.452–1.385	0.412
HFL+ONL	0.897	0.813–0.990	**0.031**	0.868	0.738–1.020	0.085
MEZ	0.847	0.668–1.073	0.169	1.229	0.912–1.657	0.176
OS	0.851	0.759–0.953	**0.005**	0.964	0.791–1.175	0.715
IZ+RPE	0.921	0.753–1.126	0.422	1.681	1.149–2.461	0.052

*P_1_ and P_2_ were calculated by multivariate logistic regression analysis between WMH and HC, PD and HC groups, respectively*.

*OR represents the odd ratio and 95% CI denotes the 95% confidence interval*.

## 3. Discussion

The retina is an extension of the brain and central nervous system. Some lesions in the brain will produce corresponding changes in some areas of the retina. Therefore, the retina is an ideal place to study Parkinson's Disease, Alzheimer's Disease, stroke and other brain diseases. Identifying early, non-invasive and inexpensive biomarkers for proxy outcome measurement has important clinical significance for the diagnosis of brain diseases. We evaluated the thickness of the outer retina in different groups, and found some sublayer thickness differences in the PD and WMH groups when compared with the HC group.

### 3.1. Parkinson's Disease and the IZ+RPE Layer of the Retina

Degeneration of dopaminergic neurons in substantia nigra is an important feature of Parkinson's Disease, and dopamine is the key neurotransmitter of motor function. Earlier studies showed that dopamine-containing neurons are found in the retina, particularly in the interamacrine cell of the inner plexiform layer and in the flexor cells of the inner and outer plexiform layers (Frederick et al., 1982). Almost all types of retinal neurons have dopamine receptors, whether in synaptic contact or not (Djamgoz et al., 1997). Most patients with PD experience symptoms of impaired vision during the course of their illness (Bodis-Wollner, 2013). In addition, dopamine was reported to induce axial eye elongation, which suggests that dopamine can prevent myopia (Papastergiou et al., 1998). These studies indicated that PD may contribute to structural and functional retinal changes on account of abnormalities in retinal dopamine.

Some prior studies have focused on changes in outer retinal layer thickness in brain-related diseases. Spund et al. (2013) found that there was no statistical difference in photoreceptor thickness in the central fovea between PD and HC groups. Roth et al. (2014) concluded that combined ONL and photoreceptor layer thickness were significantly decreased in PD patients compared to HC. Altintaş et al. (2008) reported that, compared with the control group, the mean retinal nerve fiber layer (RNFL) thickness was significantly reduced in Parkinson's disease patients.

In our study, however, some of the obtained results were inconsistent with previous studies. For instance, we found that the thickness of the IZ+RPE layer increases in the PD group (*p* = 0.052), while the thickness of the HFL+ONL/MEZ/OS layer shows no statistical difference between the PD and HC groups. On one hand, a relatively small number of samples is one of the major issues for inconsistency. On the other hand, and we also consider the differences in the instruments and embedded software used may cause inconsistencies between different studies. For example, The data used in study (Roth et al., 2014) was captured by spectral domain OCT (Cirrus HD-OCT Version 5.0, Carl Zeiss Meditec, Dublin, CA, USA), and the retinal thickness measured by commercially available OCT Model 3000 unit (Model 3000, software version A1.1, Carl Zeiss Meditec, Inc., Dublin, California, USA) in study (Altintaş et al., 2008), while the data we studied was acquired by RTVue XR Avanti SD-OCT system (Optovue Inc., Fremont, California, USA), and different imaging devices may lead to the heterogeneity in imaging retinal tissue and image quality.

RPE is the outermost cell layer of the retina whose function is to nourish the retinal visual cells. As it is reported that the RPE layer can produce levodopa, a precursor to dopamine. The RPE transplantation into the striatum might be a promising prospect in clinical treatment of PD (McKay et al., 2006; Ming et al., 2009). The increasing of RPE layer in PD is likely to be a kind of compensation mechanism in response to the shortage of dopamine. Nevertheless, further study is required for confirmation.

### 3.2. White Matter Hyperintensities and the ONL+HFL/OS Layer of the Retina

White matter hyperintensity, one of the imaging features of Cerebral Small Vessel Disease (CVD), is a clinically important marker of several common brain diseases, such as dementia and stroke. White matter is the site where nerve fibers gather in the brain, and is mainly composed of the dendrites and axons of brain neurons. Previous studies have explored the relationship between ganglion cells and white matter (Ong et al., 2015; Mutlu et al., 2017; Tak et al., 2018). Ganglion cells are located in the inner layer of the retina: nerve fiber layer (NFL), ganglion cell layer (GCL) and inner plexiform layer (IPL) together forming the ganglion cell complex. The NFL and IPL are composed of axons and dendrites respectively, and they are more likely to reflect the condition of the white matter as a whole. The GCL is composed of cell bodies, and it is more likely to be related to gray matter (Mutlu et al., 2017). Qu et al. (2020) has also recently reported that the thickness of the NFL and GCL+IPL were associated with WMH, and deteriorate with the severity of lesions.

To the best of our knowledge, no effort has been made to unveil any correlation between outer retinal layer thickness measurements between WMH and HC groups. In our study, we found that the INL+HFL and OS thickness of the WMH group was significantly thinner than in the HC group. Interestingly, the INL+HFL contains bundles of unmyelinated cone and rod photoreceptor axons (Lujan et al., 2011), whereas the OS is equivalent to the dendritic part of a photoreceptor cell. The decrease of the thickness of these two layers is consistent with the findings of a prior study of ganglion cells, in which the sublayer of axons and dendrites became thinner (Qu et al., 2020). This provides new evidence for a correlation between the neurites of retinal cells and white matter.

We speculate that the concentration of amyloid beta protein(Aβ) may lead to changes in the neurite layer of retinal neurons. Aβ is one of the important causes of Alzheimer's disease, and it is also related to WMH (Osborn et al., 2018). Animal experiments have shown that the concentration of Aβ is positively related to synaptic activity. Thus, it is reasonable to speculate that Aβ deposition may lead to the degeneration of different nerve cells in the retina, resulting in the thinning of the related sublayers. Unfortunately, our data did not record the amyloid beta protein content of patients and the control group, but this provides a specific idea for follow-up research.

### 3.3. Limitations

Our study still has some limitations. On one hand, this study lacks longitudinal follow-up. Our WMH group might contain a mixture of AD patients, dementia subjects with Lewy bodies, and stroke participants. It should be split up into several subgroups to better understand the different clinical relationship. On the other hand, our present study is also limited by the relatively small sample size of the PD group. Hence, expansion of the dataset pool is desirable.

## 4. Conclusion

In conclusion, we used a state-of-the-art deep learning-based method to segment the outer retinal layers, and further measure the thickness of the sublayers. We found that retinal degeneration in the ONL+HFL and OS were independently associated with the WMH, and that the thickness of the IZ+RPE in the PD group was significantly greater than in the HC group, providing new evidence that some brain diseases may cause changes in the retina. Finally, we recommend that future studies should expand the sample size and employ longitudinal designs to further elucidate the relationship between the WMH/PD and outer retinal thickness.

## Data Availability Statement

The raw data supporting the conclusions of this article will be made available by the authors, without undue reservation.

## Ethics Statement

The studies involving human participants were reviewed and approved by Ningbo Institute of Materials Technology and Engineering, Chinese Academy of Sciences. The patients/participants provided their written informed consent to participate in this study.

## Author Contributions

YZ and JinZ were involved in data analysis and interpretation and drafting and revising the manuscript. BC, JG, JX, QY, and YM were involved in data analysis. YW was involved in data interpretation. YG, JioZ, and JL were involved in study conceptualization, supervision, and revising the manuscript. All authors contributed to the article and approved the submitted version.

## Funding

This work was supported in part by the Shenzhen Natural Science Fund (JCYJ20200109140820699 and the Stable Support Plan Program 20200925174052004), Zhejiang Provincial Natural Science Foundation of China (LZ19F010001), in part by the Youth Innovation Promotion Association CAS (2021298), in part by the Key Research and Development Program of Zhejiang Province (2020C03036), and in part by the Ningbo 2025 S&T Megaprojects (2019B10033 and 2019B1006).

## Conflict of Interest

The authors declare that the research was conducted in the absence of any commercial or financial relationships that could be construed as a potential conflict of interest.

## Publisher's Note

All claims expressed in this article are solely those of the authors and do not necessarily represent those of their affiliated organizations, or those of the publisher, the editors and the reviewers. Any product that may be evaluated in this article, or claim that may be made by its manufacturer, is not guaranteed or endorsed by the publisher.
