# Supplementary material for: Outer Retinal Layer Thickness Changes in White Matter Hyperintensity and Parkinson's Disease
Source: Front Neurosci. 2021 Sep 14;15:741651. doi: 10.3389/fnins.2021.741651 (PMC8477009; doi:10.3389/fnins.2021.741651)
Supplement: Supplementary file 1 [file Data_Sheet_1.docx]

**APPENDIX**

The CE-Net (Gu et al., 2019) consists of 3 main components: a pre-trained feature encoder, a context extractor and a feature decoder. The feature encoder adopts pre-trained ResNet-34 as the backbone, which retains the first four feature extraction blocks without the global average pooling layer and the fully connected layers. Compared with the encoder blocks in U-Net, the ResNet block introduces a residual skip connection, which is advantageous to avoid gradient vanishing, and is able to accelerate the training procedure. The context extractor is composed of a dense atrous convolution (DAC) block and a residual multi-scale pooling (RMP) block. The DAC module consists of 4 cascaded branches with 3, 7, 9, and 19 respectively as the receptive field. One 1 × 1 convolution followed by ReLU is applied at the end of each convolution block. Finally, feature maps from the four cascaded branches and the input of the DAC module are added together as output.

The RMP module contained 4 pooling kernels of different sizes: 2 × 2, 3 × 3, 5 × 5, and 6 × 6, and outputs feature maps of various sizes. After pooling layer, a 1 × 1 convolutional layer is used to reduce the feature dimension to the 1/N of the original dimension, where N denotes the channel number of the original feature. The low-dimensional feature map is then rescaled to the same size as the original feature via an upsampling layer. Finally, the original features are concatenated with the upsampled features.

The feature decoder consists of 4 convolution blocks, and is used to restore the high-level semantic features extracted from the feature encoder. Each block includes two 1 × 1 convolution layers and a 3 × 3 transposed convolution layer. Skip connection is adopted to transmit high-resolution information from the encoder to the decoder to compensate for information reduction, and the transposed convolution layer is applied to obtain high-resolution features. Based on the skip connection and transposed convolution operation, the feature decoder outputs a prediction mask of the same size as the original image. The complete network framework is shown in Fig. 5.
